# Ribosomal proteins as unrevealed caretakers for cellular stress and genomic instability

**DOI:** 10.18632/oncotarget.1784

**Published:** 2014-02-18

**Authors:** Tae-Hyung Kim, Patrick Leslie, Yanping Zhang

**Affiliations:** ^1^ Department of Radiation Oncology, University of North Carolina, Chapel Hill, NC, USA; ^2^ Lineberger Comprehensive Cancer Center, University of North Carolina, Chapel Hill, NC, USA; ^3^ Curriculum in Genetics and Molecular Biology School of Medicine, University of North Carolina at Chapel Hill, Chapel Hill, NC, USA; ^4^ Laboratory of Biological Cancer Therapy, Xuzhou Medical College, Xuzhou, China

**Keywords:** Ribosomal Protein, Mdm2, p53

## Abstract

Ribosomal proteins (RPs) have gained much attention for their extraribosomal functions particularly with respect to p53 regulation. To date, about fourteen RPs have shown to bind to MDM2 and regulate p53. Upon binding to MDM2, the RPs suppress MDM2 E3 ubiquitin ligase activity resulting in the stabilization and activation of p53. Of the RPs that bind to MDM2, RPL5 and RPL11 are the most studied and RPL11 appears to have the most significant role in p53 regulation. Considering that more than 17% of RP species have been shown to interact with MDM2, one of the questions remains unresolved is why so many RPs bind MDM2 and modulate p53. Genes encoding RPs are widely dispersed on different chromosomes in both mice and humans. As components of ribosome, RP expression is tightly regulated to meet the appropriate stoichiometric ratio between RPs and rRNAs. Once genomic instability (e.g. aneuploidy) occurs, transcriptional and translational changes due to change of DNA copy number can result in an imbalance in the expression of RPs including those that bind to MDM2. Such an imbalance in RP expression could lead to failure to assemble functional ribosomes resulting in ribosomal stress. We propose that RPs have evolved ability to regulate MDM2 in response to genomic instability as an additional layer of p53 regulation. Full understanding of the biological roles of RPs could potentially establish RPs as a novel class of therapeutic targets in human diseases such as cancer.

## INTRODUCTION

The ribosome is responsible for the translation of genetic information from mRNA to protein in all living cells. Translation has gained attention as a potential anti-neoplastic target because cancer usurped those pathways [1]. The ribosome is composed of two subunits consisting of a complex of ribosomal RNAs (rRNAs) and ribosomal proteins (RPs) (reviewed by Melnikov et al. [2]). In eukaryotic ribosomes, 40S and 60S subunits associate to form the translationally active 80S ribosome. Four rRNAs (25S, 5.8S, and 5S in the large subunit and 18S in small subunit) and one molecule each of about eighty different RPs are required to assemble the fully active ribosome in eukaryotes [3]. Ribosomal proteins, which compose half of the ribosome, have recently been highlighted because of their roles in the regulation of p53 via binding to MDM2. The main purpose of this review is to discuss the potential advantages in maintaining the binding of multiple distinct RPs to MDM2.

## RIBOSOMAL PROTEINS AND THEIR REGULATION

Ribosomal proteins are expressed in abundance in metabolically active cells undergoing protein synthesis. The estimated average number of ribosomes per *E. coli* cell is 10,000, which accounts for approximately 15% of the total cellular protein content. Metabolically active eukaryotic cells are estimated to harbor approximately 10^6^-10^7^ ribosomes (5-10% of total cellular proteins), which are required to meet the high demand for protein synthesis [4-6]. When cells proliferate in the presence of mitogenic stimuli such as growth factors or nutrients, cells up-regulate ribosome biogenesis and protein synthesis to fulfill the requirements of cell growth and proliferation. Not surprisingly, ribosomal protein expression is directly linked to cell cycle progression, and initial evidence for this link arose from a conditional knockout mouse study of ribosomal protein S6 (RPS6). Mouse hepatocytes lacking RPS6 completely lost their regenerative capacity following a partial hepatectomy due to apparent disruptions in the cell cycle [7]. Furthermore, phosphorylation of RPS6, which has been implicated in regulation of translation and cell growth, can be inhibited when cells are treated with nutlin-3a, an MDM2 inhibitor, though the underlying mechanism of the inhibition has not been elucidated [8, 9]. Another line of evidence came from genetic studies analyzing the fruit fly *Drosophila melanogaster*. Defects in ribosomal protein production and consequently defects in ribosome biosynthesis have been linked to the *Minute* phenotype through the identification of 64 *minute* loci in *Drosophila* [10-16]. Deregulation of ribosomal protein production and ribosome dysfunction has also been implicated in many human diseases such as Diamond-Blackfan anemia, 5q syndrome, Shwachman-Diamond syndrome, X-linked dyskeratosis congenita, cartilage-hair hypoplasia, and Treacher-Collins syndrome (See details in [17] and [18]). In addition to RP haploinsufficiency, overabundance of certain RPs is another form of ribosomal stress that can disrupt the delicate balance of RPs required for ribosomal biogenesis. Indeed, both eukaryotic and prokaryotic cells have developed feedback mechanisms to regulate the expression level of RPs. In prokaryotic cells, RPs can self-regulate their synthesis by various autoregulatory transcriptional and translational mechanisms [19-22]. Such autoregulation is well conserved in eukaryotic cells as a pathway to dispose of excess RPs [23-31]. These evolutionarily conserved RP surveillance systems allude to the importance of cellular self-evaluation regarding ribosomal protein production [18, 32]. Deregulated over- or under-expression of some RPs can result in the accumulation of other RPs. Once a cell registers imbalances in RP production, the cell eliminates excess RPs because equimolar production of rRNA and RPs is crucial for proper ribosome assembly [27, 33-35]. Ribosome biogenesis has been linked to p53 stability [36]. Imbalances in RPs can induce RP dosage-dependent p53 activation via MDM2. CDKN2A (also known as p14^ARF^ /p19^Arf^) and Ataxia Telangiectasia Mutated (ATM) kinase suppress MDM2 in the presence of oncogenic and genotoxic stresses, respectively. CDKN2A inhibits MDM2 function by directly binding to MDM2, whereas ATM suppresses MDM2 through post-translational modifications [37]. Similarly, RPs have recently been shown to directly bind to MDM2 in response to nucleolar stress resulting in the negative regulation of MDM2 and the subsequent activation of p53 (for a detailed review, see [38, 39]. Therefore, the tight regulation of RP and rRNA expression is important not only for normal cell physiology but also to avoid the activation of RP extraribosomal functions.

## EXTRARIBOSOMAL FUNCTIONS OF RPS

Ribosomal proteins play important roles in diverse cellular physiological processes in addition to their basic roles in protein synthesis. In 1974, the first extraribosomal function of RPs was proposed based on the observation that bacteriophage Qβ expresses a peptide that interacts with three host (*E. coli*) proteins (EFTu, EFTs, and RPS1) and functions as an RNA replicase used for the replication of the phage genome. RPS1 binds to specific regions of the Qβ peptide and regulates the function of the Qβ genome (reviewed in [40]). Since the first report of extraribosomal functions of RPS1 in *E. coli*, additional extraribosomal functions of various RPs have been revealed by knockdown or knockout of RPs in various animal models. Knockdown of RPS19 in zebrafish leads to anemia [41, 42], and even more strikingly, knockdown of RPL11 in zebrafish results in developmental defects in the head [43]. Mutations in RPS19 or RPS20 in mice result in abnormal melanocyte proliferation and red blood cell hypoplasia [44]. RPL22 inactivation promotes cell transformation by inducing expression of the stemness factor Lin28B in mice [45]. Mutations in RPS19 are also associated with 25% of Diamond-Blackfan anemia cases (DBA). Currently, nine RPs have been implicated in DBA (RPL5, RPL11, RPL35A, RPS7, RPS10, RPS17, RPS19, RPS24, and RPS26) [46, 47]. In humans, RPS14 haploinsufficiency leads to 5q syndrome [48], and RPS4 haploinsufficiency appears to be responsible for Turner syndrome in females [49]. Collectively, these observations strongly suggest that imbalances in certain ribosomal proteins result in various detrimental phenotypes related to the extraribosomal functions of RPs.

## THE NUCLEOLUS AS A CENTER FOR THE CELLULAR STRESS RESPONSE

The nucleolus is a subnuclear non-membrane-bound structure where ribosome biogenesis occurs. Translocation of ribosomal proteins from the nucleolus to the nucleoplasm due to defects in ribosome biogenesis can trigger a stress response associated with nucleolar stress. Considering that most types of cellular stress are associated with the disruption of nucleolar integrity, which results in the release of ribosomal proteins from the nucleolus to the nucleoplasm, the nucleolus could conceivably function as a structure that is integrally involved in stress sensing [50]. In other words, the integrity of the nucleolus, as indicated by the fidelity of ribosome biogenesis, appears to represent a mechanism by which the cell can gauge whether the cellular conditions are favorable for growth and proliferation. Chemical reagents (actinomycin D, 5-fluorouracil, and mycophenolic acid) or cellular events that disrupt steps involved in ribosome biogenesis (inhibition of rRNA synthesis, impaired rRNA processing, and RP imbalances) can cause nucleolar stress (reviewed in [51, 52]). For example, upon release from the nucelolus, RPL26 interacts with Nucleolin to enhance p53 translation following DNA damage [53]. As a result of RP concentration imbalances in the presence of stress, most, if not all, RPs capable of binding MDM2 are released from the nucleolus. Upon release, the RPs bind MDM2 and cause p53 accumulation via the stabilization of p53 and/or an increase in p53 translation, which ultimately results in cell cycle arrest or apoptosis. As a result, the stress response initiated in the nucleolus becomes transmitted to the p53 pathway through RP-MDM2 binding.

## MDM2 IS REGULATED BY A FLOCK OF RPS

**Figure 1:**
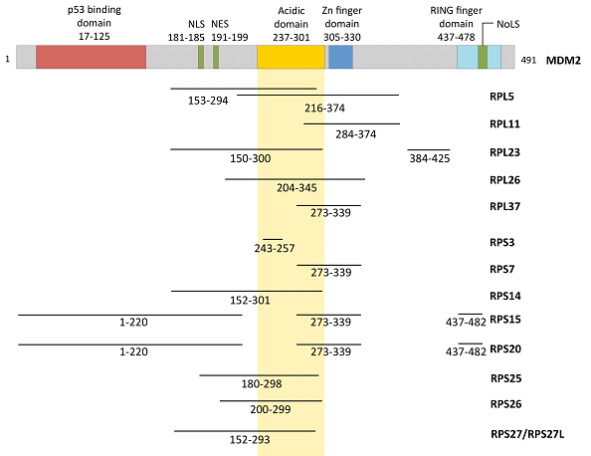
Functional domains of MDM2 and the regions that are bound by various RPs

MDM2 is the best characterized negative regulator of p53. MDM2 is an E3 ubiquitin ligase that encodes a C-terminal RING domain and has multiple protein-binding domains including a p53-binding region at the N-terminus and an MDMX (also known as MDM4)-binding region through the RING domain. In addition, the acidic domain and the zinc finger domain in the central region of MDM2 have been shown to interact with a variety of regulatory factors such as the tumor suppressor p14^ARF^ and multiple ribosomal proteins [55] (Figure 1). To date, fourteen RPs (L5, L11, L23, L26, L37, S3, S7, S14, S15, S20, S25, S26, S27, and S27L) have been shown to bind directly to MDM2 (Table 1).

**Table 1:** List of MDM2-binding RPs

MDM2-Binding RPs	Interacting residues in MDM2	Chromosomal location in human (mouse)	References
Large Subunit			
L5	153-294, 216-374	1p22.3-p22.1 (5)	[98, 99]
L11	284-374	1p36.1-p35 (4)	[100, 101]
L23	150-300, 384-425	17q11.2-q12 (11)	[101-103]
L26	204-345	17p13 (11)	[104, 105]
L37	273-339	5p13.3-p13.1 (15)	[106, 107]
Small Subunit			
S3	243-257	11q13.3-q13.5 (7)	[108]
S7	273-339	2p25.3-p25.1 (12)	[109, 110]
S14	152-301	5q33.1-q33.3 (18)	[59]
S15	1-220, 273-339, 437-482	19p13.3 (10)	[106]
S20	1-220, 273-339, 437-482	8q11.2-q13 (4)	[106, 111]
S25	180-298	11q23.3 (9)	[112]
S26	200-299	12q13 (10)	[113]
S27	151-293	1q21.1-21.3 (3)	[114]
S27L	151-293	15q22.1 (9)	[114]

## CHARACTERISTICS OF THE RP-MDM2 INTERACTION

MDM2-binding RPs lack obvious similarities in amino acid sequences or three-dimensional structures [56, 57]. However, most MDM2-binding RPs share at least one feature in that they bind to the central acidic domain (CAD) of MDM2 (amino acid residues 237-301) (Figure 1). Electrostatic interaction appears to be one of the major binding forces between the MDM2 CAD and the basic residues within the MDM2-binding proteins. Most RPs are very basic proteins with pI (isoelectric point) greater than 10, which is consistent with their innate functions as RNA-binding proteins [18]. Interestingly, the most well known MDM2 regulator p14^ARF^/p19^Arf^ is also highly basic (pI>12), which supports the idea that electrostatic interaction plays a major role in binding the MDM2 CAD [58]. However, even though electrostatic interactions can at least partially explain why MDM2-binding RPs interact with the CAD of MDM2, additional mechanisms that determine the selectivity of the RP-MDM2 interaction probably exist. Furthermore, because so many RPs can bind to MDM2 and because many of the RP binding sites overlap, one may predict that the order of RP binding is tightly regulated. One possible mechanism of RP-MDM2 specificity that merits further investigation is the role of post-translational modifications within the RP binding regions. Indeed, many of the RP binding sites within MDM2 contain residues that are phosphorylated and rendered negatively charged by various kinases such as the serine/threonine kinase casein kinase I [54]. Studies investigating the effect of various MDM2 post-translational modifications on the many RP-MDM2 interactions could yield valuable insight into MDM2 regulation. Despite the fact that 14 RPs have been shown to bind MDM2, approximately 80 basic RPs exist, which suggests that the total number of RPs found to bind MDM2 will likely increase over the next few years. In light of the multitude of reports confirming and expounding on RP-MDM2 interaction, a fundamental question remains: why do so many ribosomal proteins interact with MDM2?

## WHY DO MULTIPLE RPS INTERACT WITH MDM2?

Recently, Zhou et al. briefly discussed some reasons why mammalian cells have conserved the ability of so many RPs to activate p53 via MDM2 [59]. The authors hypothesized that individual RPs are required to work together to efficiently inactivate MDM2. Alternatively, the authors proposed that each MDM2-binding RP may bind to MDM2 at different time points in a sequential manner depending on which RPs are released earlier following stress. Lastly, the authors speculated that different types of stress may inhibit MDM2 through different RPs. In the following section, we further expand the discussion.

### (1) Multiple RPs bind to MDM2 to produce a stronger effect than a single RP

As one might expect, RPs can interact with each other and with other rRNAs, as RPs require these interactions to assemble a fully functional ribosome. For example, RPL7 and RPL12 in *E. coli* form a functionally important domain in the ribosome (reviewed in [60]). In addition to the formation of ribosomes, several studies have reported that some RPs interact with each other to execute certain extraribosomal functions. For example, in bacteria, RPS1 and RPS2 interact with each other to initiate translation [61]. Although RPL5 and RPL11 can suppress MDM2 individually, previous reports have suggested that the protective effect of RPL5 and RPL11 on p53 stability is even more evident when both proteins are present [62]. It will be interesting to determine whether RPL5 (or RPL11) synergizes with other RPs when they bind MDM2 simultaneously. The synergistic suppression of MDM2 by the concomitant binding of multiple RPs may result in greater induction of p53.

### (2) Different types of stress might cause imbalances in RP production and induce the binding of specific RP(s) to MDM2

Different MDM2-binding RPs may be present in the nucleoplasm in different concentrations depending on the specific type of ribosomal stress signal. Considering the fact that all RPs share the purpose of assembling the ribosome, it is unlikely that MDM2-binding RPs can sense distinct stressors individually. Nevertheless, this hypothesis cannot be ruled out based on the fact that most stress types including DNA damage, temperature change, hypoxia, alteration of proteasome activity, viral infection, oncogenic stress, and transcriptional inhibition cause the disruption of the nucleolus, which activates the nucleolar stress response (reviewed in [52]). Various agents that mimic these stresses have been identified, and all of the aforementioned agents induce p53 stabilization following nucleolar disruption. These stresses are also associated with the covalent modification of p53. However, the mechanism by which p53 integrates such a wide range of stimuli remains unknown [50, 52]. Recent studies have shed light on the pivotal roles of RPs in p53-induced stress responses via MDM2 regulation (reviewed in [38]). Therefore, it is possible that different stresses may cause differential expression of individual RPs through mechanisms ranging from transcription regulation, protein stability, transport, assembly variations, etc. Many RPs may have individually evolved the ability to regulate MDM2 in response to a particular type of stress. This “RP and stress pair” could integrate many different types of stress into a p53-mediated response. It is worth noting that many characterized RP-MDM2 interactions are based on *in vitro* cell culture systems. Therefore, the roles of many MDM2-binding RPs in p53 regulation remain to be confirmed *in vivo*.

### (3) Complex of multiple RPs might be required to regulate MDM2

The fact that several RPs can interact with MDM2 may imply that single RP binding is not sufficient to suppress MDM2. In other words, the formation of a complex between multiple RPs and MDM2 may not have a synergistic effect (as discussed above) but may be a minimum requirement for MDM2 regulation. If this is the case, then individual RPs would need to coordinate as a sub-ribosomal complex to bind to multiple MDM2 molecules, as MDM2 appears to function as a multi-subunit complex [59]. One study has reported evidence that ribosomal proteins directly interact with each other to negatively regulate MDM2 [62]. In this study, RPL5 and RPL11 were shown to physically interact with each other via 5S rRNA resulting in additional repression of MDM2. In addition, complex formation with other co-factors has been proposed as another layer of ribosome regulation. RPL5 and RPL11 appear to be required to form a complex with assembly factors Rpf2 and Rrs1 for proper RNA processing [63]. Nevertheless, some RPs, such as RPS19, have been shown to interact with more than 150 other proteins including 25 ribosomal proteins (14 RPs in the 40S ribosomal subunit and 11 RPs in the 60S subunit) in human erythroleukemia K562 cells [64]. Intriguingly, RPS19 has a wide variety of proteins that comprise its interactome, which consisted of NTPases (ATPases and GTPases), kinases, and transcription factors.

## RPL11 IS AN IMPORTANT PLAYER IN P53 ACTIVATION

Among the many MDM2-binding RPs, RPL11 appears to be the most important for MDM2 regulation. Recently, RPL5 and RPL11 have been shown to be protected from proteosomal degradation after ribosomal biogenesis stress, whereas other MDM2-binding RPs such as L23 L25, and S7 were not protected [65]. This study suggests that RPL5 and RPL11 play crucial roles in the RP-mediated activation of p53 and concurrently reconfirms the critical role of the nucleolus in the stress response. RPL11 was one of the first RPs to be characterized as a major stress-responding RP. Inhibition of ribosome biogenesis results in the increased expression of RPL11, which inhibits MDM2-mediated p53 ubiquitination [66]. Recently, physical and functional interaction between RPL11 and p14^ARF^ has been reported [67]. This study demonstrated that p14^ARF^ induces ribosomal stress, which causes the up-regulation of RPL11 and subsequently results in stronger MDM2 inhibition. At the transcriptional level, the RPL11-MDM2 complex has been shown to directly localize to the promoter region of p53 target genes and activate those genes [68]. In another study, three (L5, L11, and L26) out of fourteen MDM2-binding RPs were reported to interact with Pict1, a novel MDM2-p53 pathway regulator. Sasaki et al. carried out siRNA-mediated knockdown against four MDM2-binding RPs (RPL5, RPL11, RPL23, and RPS7), and found that RPL11 is the only RP that is crucial for the inhibition of MDM2 function in doxycycline-treated mouse ES cells [69].

## STRUCTURAL FEATURES OF RPL11

Structural features of RPL11 have also indicated the potential for unique roles of RPL11 regarding ribosomal and extraribosomal functions. A high-resolution structure of the eukaryotic ribosome has recently been published [70]. Prior to this study, the same research group described the crystal structure of the eukaryotic ribosome, and they indicated that RPL11 and RPL5 are located in the central protuberance of the large subunit. Furthermore, RPL11 appears to be in contact with the head domain of the 40S subunit, an arrangement that is well conserved in prokaryotic ribosomes as well [71]. In prokaryotes and eukaryotes, the ribosome translates proteins through the use of a ratcheting movement between the small and large subunits. For the ratcheting movement of ribosomes in eukaryotes, the contact points between the two subunits known as the “bridge” are critical. Therefore, RPL11 seems to play a crucial role in the constant adjustment of the bridge as the ratcheting movement progresses. Another interpretation can be made based on those ribosomal structure studies. As mentioned above, RPL5 and RPL11 can directly interact with 5S rRNA [62, 71]. The transcription and processing of rRNA, which occur in the nucleolus, are sensitive to different types of cellular stress [52]. Because RPL11 and RPL5 co-localize and interact with rRNA directly, these two RPs may function as the main sensors for problems that arise in ribosome biogenesis (i.e. rRNA transcription or processing) that may result from different types of cellular stress. This interpretation is consistent with the aforementioned statement that RPL11 appears to be the most important player in p53 activation and stress sensing.

## REGULATION OF OTHER PROTEINS BY RPL11

MDMX is known to play a role in p53 regulation via its ability to bind to both p53 and MDM2 [72]. Although MDMX has no intrinsic E3 ligase activity and does not promote p53 degradation, MDMX exhibits considerable homology to MDM2 and forms a stable heterodimer with MDM2. The interaction between MDM2 and MDMX is well known to enhance the ability of MDM2 to function as an E3 ubiquitin ligase to degrade p53, although the associated mechanism remains unclear [73-78]. Gilkes et al. reported that RPL11 promotes MDMX degradation by binding to MDM2. In this study, the authors ectopically expressed RPL11 in the HCT116 p53^−/−^ cell line and found that whereas overexpressed RPL11 did not affect MDM2 protein expression, it stimulated MDMX polyubiquitination by MDM2 [79]. It is worth to note that the increased E3 ligase activity of MDM2 on MDMX upon RPL11 binding is contradictory to the finding that RPL11-MDM2 interaction results in decreased polyubiquitination and increased stability of p53. Binding of RPL11 to MDM2 may skew MDM2 E3 activity toward MDMX rather than p53 via conformational change of MDM2. Alternatively, RPL11-MDM2 interaction generally increases MDM2 E3 activity, but simultaneous binding by other RPs to MDM2 may direct the E3 activity towards MDMX. Additionally, RPL11 has been shown to inhibit the cell cycle via the c-Myc pathway. RPL11 binds to c-Myc and inhibits the expression of c-Myc target genes by recruiting the key coactivator transformation-transactivation domain-associated protein (TRRAP) to the c-Myc promoter regions. The c-Myc mRNA level is also regulated by RPL11 via the recruitment of miR-24/miRISC to the 3' untranslated region of c-Myc mRNA [80, 81]. Interestingly, RPL11 is one of the transcriptional targets of c-Myc, which implies the existence of a c-Myc negative feedback loop [82]. c-Myc is a major regulator of protein synthesis and ribosome biogenesis (reviewed in [83]). Because RPL11 and c-Myc form a potential negative feedback loop, it is plausible that RPL11 plays a critical role in the biogenesis of ribosomes and RPs themselves. Taken together, one may reasonably conclude that RPL11 is the most critical RP responsible for the ribosomal and extraribosomal functions of RPs. Increasing attention has been given to the RPL11-MDM2-p53 pathway. Recently, Olausson et al. reviewed several novel regulators of the RPL5/RPL11-MDM2-p53 pathway [84], therefore, the regulatory mechanisms associated with this pathway appear to be key to a better understanding of overall p53 regulation.

## CAN RPS FUNCTION AS SENSORS FOR ANEUPLOIDY?

**Figure 2:**
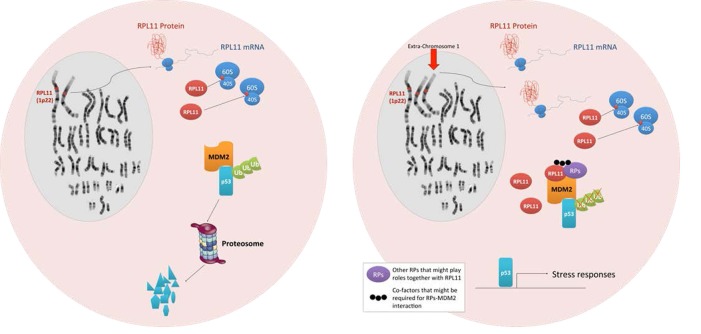
Models depicting aneuploidy-based imbalances in RPs that result in the activation of the stress response via MDM2 binding and p53 activation Left, a cell with a normal karyotype produces equal proportions of RPs and does not activate p53. Right, an aneuploid cell with an extra copy of chromosome 1 (indicated by an arrow) expresses a disproportionate amount of RPL11 resulting in the activation of the p53 stress response pathway.

An important topic that has not yet been discussed thus far is the fact that genes encoding RPs are widely dispersed over many different chromosomes. In humans, all 80 RP genes are found on both sex chromosomes and 20 autosomes (only chromosomes 7 and 21 do not encode RP genes), whereas the 4 rRNA genes are clustered in 6 autosomes [85]. The RPs that have been reported to bind to MDM2 are also distributed among nine different autosomes in humans and ten different autosomes in mice (Table 1). Due to the dispersion of RP genes throughout the human genome, any total or partial loss of chromosomes (e.g. monosomies and partial monosomies) is likely to result in heterozygous deficiencies of one or more RP genes, and such aneuploidy may account for the abnormal development and poor viability of monosomic human fetuses [86]. If this is the case, then imbalances in RPs due to aneuploidy may play a key role in several human diseases that are commonly associated with aneuploidy such as cancer. The determination of how cells respond to aneuploidy is a major focus of cancer research. Despite the dosage compensation effect, recent studies have shown that chromosome copy number change generally results in changes in protein abundance in yeast [87]. Yet, it is unclear whether aneuploidy causes transcriptional and protein expression changes within the cell in a manner that correlates with the DNA copy number (reviewed in [88]). Nevertheless, aneuploidy has been correlated with the induction of the p53 response [89]. In normal cells, aneuploidy that occurs following chromosome missegregation, which induces DNA damage on the lagging chromosomes, or stresses such as proteotoxic stress and metabolic stress results in p53 activation via ATM [90, 91]. Numerical and structural chromosomal abnormalities are also observed in the vast majority of cancer genomes. Although whether such aneuploidy is a cause or a consequence of malignant transformation remains unclear, numerous lines of evidence indicate that aneuploidy can predispose cells to malignant transformation. However, the underlying mechanisms that drive tumorigenesis remain to be identified [88]. Recently, a large-scale DNA copy number analysis in human cancers revealed that an average of 25% of the cancer genome displays some form of aneuploidy [92]. Consistently, analysis of the Mitelman Database has also shown whole-chromosome alterations that occur in several cancer types [88]. If aneuploidy triggers a common stress response, then all aneuploidic cancer cells would presumably require specific adaptations to allow proliferation in the presence of their abnormal genomes. Should cancer cells share common mechanisms that enable them to tolerate aneuploidy by avoiding the stress response, these mechanisms could then represent novel therapeutic targets. Considering the large number of RP genes, one may reasonably conclude that RP genes are largely dispersed among the genome as a reflection of the early origin of these genes during evolution. However, the dispersion of the RPs throughout the genome simultaneously offers the cell an inherent mechanism through which it can recognize most genomic abnormalities, which are manifested in the deregulation of RP expression. Thus, RPs can play a crucial role in normal cells to protect against aneuploidy (Figure 2). Given that the RP genes are encoded on virtually all of the human chromosomes, an imbalance in RPs due to aneuploidy may work as an additional safeguard through which p53 can be activated. In other words, the differential expression of RPs in stressed cells due to increased genomic instability or aneuploidy may serve as an alarm system (or a novel stress sensor) to induce p53 activation through the suppression of MDM2.

In addition to p53-dependent roles of RP-MDM2 interactions in responding to genome instability, evidence suggests that p53-independent roles may also exist. For example, the Eischen group has reported that MDM2 overexpression results in a p53-independent increase in genome instability by binding to the DNA damage repair protein Nbs1 [93]. Furthermore, MDM2 transgenic mice expressing elevated levels of MDM2 display increased genome instability presumably due to increased binding of Nbs1 by MDM2 [94]. Interestingly, Nbs1 binds MDM2 amino acids 198–228, which overlaps with the binding site for at least eight RPs (RPL5, RPL23, RPL26, RPS14, RPS25, RPS26, and RPS27/2L) (see Table 1). Therefore, possible implications for p53-independent effects of RP-MDM2 interactions could involve the masking of the Nbs1 binding site in MDM2 by these RPs as a result of genome instability. Nevertheless, this potential role for RP-MDM2 interaction and the potential role of the RP genes as genomic instability sensors remain to be tested experimentally.

## CONCLUSIONS AND PERSPECTIVES

We have discussed that RPs have extraribosomal functions, and the release of RPs from the nucleolus to the nucleoplasm following stress plays important roles in p53 induction. Fourteen RPs have been shown to bind to the acidic domain and/or zinc-finger domain of MDM2, which results in MDM2 suppression and p53 stabilization. We have suggested three hypotheses that could explain why such a large number of RPs have evolved the ability to regulate MDM2. RPs may have a synergistic effect when multiple RPs bind to MDM2 simultaneously, or complex formation by multiple RPs may be required for the regulation of MDM2. Another possible scenario is that each RP responds to a distinct type of stress as a way for the cell to integrate many different types of stress into one stress response (p53 activation). No matter what the true mechanism is, it implies that those RPs play important roles in cellular quality control by regulating MDM2-p53 pathway. Occasionally, cells encounter severe stress such as DNA damage. In that case, cells have developed defense mechanisms to cope with those stresses. What about daily routine mild stresses such as nutrient stress? It is likely that cells have evolutionary developed another defense mechanisms as well against the mild and chromic stresses. We propose that RPs-MDM2-p53 pathway might play a central role in routine control of cellular quality. Among the RPs that bind MDM2, we speculate that RPL11 is the most important one with respect to its ribosomal and extraribosomal functions. RPL11 has significant roles in MDM2 and MDMX regulation, as RPL11 cooperates with p14^ARF^, RPL5, c-Myc, and other co-factors. Furthermore, RPL11 has a unique role in the ribosome structure and ratcheting movement of the two ribosomal subunits. MDM2 mutations in the region that binds to RPL11 have discovered in human cancer [95, 96]. Findings of those cancer-associated mutations in MDM2 and investigation of their roles in tumorigenesis *in vivo* further strengthen the importance of RP-MDM2-p53 pathway [39]. Finally, we posited that widely dispersed RPs in the human genome could work as a stress sensor for genomic instability because aneuploidy can result in an imbalance in RP expression, which could consequently result in the induction of p53. The importance of RPs regarding their extraribosomal functions especially in the stress response has only recently been widely accepted by the field. However, due to our limited knowledge on the functions of RPs in an extraribosomal context, a comprehensive understanding of the ribosomal protein world is far from complete. Careful and thoughtful follow-up studies will shed more light on our understanding of these multifaceted proteins. As we gain more knowledge on the physiological functions of RPs especially MDM2-binding RPs, we will be able to paint a more precise picture regarding how these RPs regulate the p53-mediated stress response. During the preparation of this review, Berkers *et al*. published a review on the roles of p53 family members in metabolic regulation highlighting the importance of p53 regulation in various metabolic diseases [97]. Ultimately, a better understanding of the RP-MDM2 mechanism of p53 regulation following stress such as nutritional stress should invariably identify novel targets that can be exploited for therapeutic benefits.
